# Immune cell profiling of peripheral blood in long-term testicular germ cell tumor survivors

**DOI:** 10.3389/fimmu.2025.1697087

**Published:** 2025-11-13

**Authors:** Kristina Kilikova, Andrea Mlcakova, Zuzana Sestakova, Katarina Kalavska, Kristyna Adamikova, Jana Obertova, Patrik Palacka, Katarina Rejlekova, Zuzana Sycova-Mila, Valentina De Angelis, Zuzana Orszaghova, Peter Lesko, Daniela Svetlovska, Beata Mladosievicova, Liang Cheng, Michal Mego, Michal Chovanec

**Affiliations:** 1 Department of Oncology, Faculty Hospital, Trnava, Slovakia; 2 Department of Medical Oncology, National Cancer Institute, Bratislava, Slovakia; 3 Flow cytometry Laboratory, Department of Laboratory Medicine, National Institute of Children’s Diseases, Bratislava, Slovakia; 4 2nd Department of Oncology, Faculty of Medicine, Comenius University & National Cancer Institute, Bratislava, Slovakia; 5 Translational Research Unit, 2nd Department of Oncology, Faculty of Medicine, Comenius University & National Cancer Institute, Bratislava, Slovakia; 6 Cancer Research Institute, Biomedical Research Center, Slovak Academy of Sciences, , Bratislava, Slovakia; 7 Institute of Pathological Physiology, Faculty of Medicine, Comenius University, Bratislava, Slovakia; 8 Department of Pathology and Laboratory Medicine Brown University Warren Alpert Medical School, the Legorreta Cancer Center at Brown University, and Brown University Health, Providence, RI, United States; 9 Department of Surgery (Urology), Brown University Warren Alpert Medical School, the Legorreta Cancer Center at Brown University, and Brown University Health, Providence, RI, United States

**Keywords:** flow cytometry, immune system, immunophenotype, late toxicity, testicular germ cell tumors

## Abstract

**Introduction:**

This study addresses changes in peripheral blood immune cell composition and possible late effects of curative treatment in testicular germ cell tumors (GCT) survivors.

**Methods:**

We analyzed the immunophenotype in peripheral blood obtained from 202 survivors treated at the National Cancer Institute in Bratislava by flow cytometry. The median long-term follow-up was 13 years (1-35). We divided the survivors into groups as follows: CT-chemotherapy (N = 141), RT-radiotherapy (N = 16), CTRT – chemotherapy + radiotherapy (N = 13), and the control group of patients under active surveillance - AS (N = 32).

**Results:**

Survivors treated with AS had a lower number of B-cells (mean ± standard deviation (SD) = 10.3 ± 3.5 vs 11.9 ± 4.2, p=0.04) compared to the CT group. Survivors treated with AS vs RT had a higher number of total lymphocytes (29.8 ± 7.7 vs 25.2 ± 6.3, p=0.04). In AS vs CTRT group B-cells (10.3 ± 3.5 vs 13.7 ± 5, p=0.01) and conventional dendritic cells (cDCs) (74.3 ± 11.8 vs 82.5 ± 6.7, p=0.04) showed lower numbers. Survivors treated with AS vs. ≤ 400 mg/m2 cumulative dose of cisplatin had fewer eosinophils (2.29 ± 1.5 vs 2.99 ± 1.7, p=0.03) and double-negative T-cells (DNT cells) (4.7 ± 3.4 vs 6.6 ± 6.6, p=0.04). In AS vs. ≥ 400 mg/m2, B cells counts were lower in the control group (10.96 ± 5.3 vs 12.3 ± 4.8, p=0.03); treatment with ≤ 400 mg/m2 vs. ≥ 400 mg/m2 resulted in higher counts of eosinophils (3.0 ± 1.7 vs 2.1 ± 1.7, p=0.00025) and DNT cells (6.7 ± 6.7 vs 4.9 ± 3.6, p=0.02).

**Conclusions:**

Our study demonstrates an association between both cisplatin-based chemotherapy and radiotherapy with specific immune cell populations, suggesting that these treatment modalities may exert long-term immunomodulatory effects.

## 1 Introduction

Germ cell testicular tumors (GCTs) are most commonly affecting males aged 15 to 40 years. The global incidence of these tumors is rising and varies significantly, with Western countries reporting between 3 and 12 new cases per 100,000 men annually (1).

Standard treatment for GCTs is radical orchiectomy and/or combination with cisplatin-based chemotherapy, radiotherapy, and retroperitoneal lymph node dissection (2). Although cisplatin-based chemotherapy has dramatically increased the cure rate of GCTs, it has also led to toxicity, altering the quality of life (3). Side effects of chemotherapy and late toxicities include peripheral neuropathy, hearing impairment, and chronic fatigue, among many others (4). Late toxicity is a central focus of our study investigating the long-term effects of chemotherapy and/or radiotherapy on the immune-cell profile in the peripheral blood of GCT survivors, as it remains largely unexplored. Since cisplatin-based chemotherapy has been proven to have immunomodulatory effects, this could have unfavorable outcomes years after the administration of therapy. The main concern may be the induction of an immunosuppressive environment leading to chronic inflammation and secondary malignancies.

Although the high response rate, TC survivors could develop short- and long-term morbidity. Given that TC survivors generally have a good life expectancy, their quality of life must be guaranteed (5). Late toxicity is a central focus of our study investigating the long-term effects of chemotherapy and/or radiotherapy on the immune-cell profile in the peripheral blood of GCT survivors, as it remains largely unexplored.

One area of growing interest is immunosenescence, or immune aging, which refers to the progressive functional decline of both adaptive and innate immune responses typically associated with chronological aging (5). Bourlon et al. conducted a case-control study of germ cell tumor (GCT) survivors treated with ≥3 BEP chemotherapy cycles and disease-free ≥3 months, compared with age-matched controls. The survivors showed an immunosenescent phenotype, including increased p16INK4a expression in CD3+ lymphocytes, reduced naïve T cells, and elevated memory T cells. While naïve B cells were unchanged, memory CD19+ cells were significantly reduced, suggesting altered immune function. These findings indicate possible increased infection and cancer risk, emphasizing the need for long-term immune monitoring in GCT survivors (6).

In addition to cellular immunological alterations, systemic inflammatory markers have also been explored for their prognostic significance in testicular GCTs. Among these, the neutrophil-to-lymphocyte ratio (NLR) has emerged as a widely studied parameter. Although not examined in the present work, these findings highlight the broader relevance of immune and inflammatory profiles in this disease. Sarejloo et al. investigated NLR in a systematic review comparing GCT patients and healthy controls, confirming its diagnostic and prognostic value, with significantly lower NLR observed in healthy individuals. In testicular cancer patients, an elevated NLR has been linked to adverse prognostic features, including advanced stage, metastases, contralateral tumor development, reduced survival, and poorer chemotherapy response (7). Chovanec et al. further assessed systemic inflammation using the systemic immune-inflammation index (SII), derived from neutrophil, lymphocyte, and platelet counts, and found that higher SII values correlated with advanced disease and worse outcomes (8). A retrospective study by Kölükçü, focusing on seminomatous GCTs, confirmed that increased pre-orchiectomy SII levels predicted advanced-stage disease, with each 10-unit rise in SII corresponding to a sixfold higher likelihood of extra testicular dissemination (9).

Building upon the significance of systemic inflammation, recent studies have also highlighted the prognostic role of tumor microenvironment immune markers, particularly PD-L1 expression. Cierna et al. demonstrated that while PD-1 is absent in GCTs, PD-L1 is markedly upregulated, correlating with more advanced disease and poorer survival outcomes (10). Conversely, Chovanec et al. found that high PD-L1 expression on tumor-infiltrating lymphocytes was associated with better progression-free and overall survival (11), suggesting that immune activation within the tumor microenvironment may confer a protective effect. Lobo et al. investigated both CTLA-4 and PD-L1 expressions in immune cells and reported that CTLA-4 overexpression correlated with favorable prognostic features, including the absence of lymphovascular invasion and lower pathological tumor and nodal stages (12).

These findings highlight the prognostic potential of systemic immune markers in GCTs and complement the immunophenotypic analyses presented in the current study. Recent evidence suggests that baseline immune depletion and immune masking may influence both tumor manifestation and tumor progression. Li Y. et al. have studied the mechanisms of invasion and metastasis, and suggest depletion of certain cells, for example myeloid cells/neutrophils/tumor-associated macrophages indirectly restores NK and cytotoxic T-cell function, counteracting tumor immune evasion. When it comes to masking, tumor cells use multiple mechanisms, for example physical cloaking within the platelet aggregates, obscuring them from immune surveillance (13). Specifically, in GCTs, Leathlobhair et al. suggest HLA loss of heterozygosity may enable immune evasion especially in subset of seminomas (14).

This study´s aim is to assess long-term immunophenotypic alterations in testicular GCT survivors across different treatment modalities to elucidate the lasting immunomodulatory effects of cisplatin-based chemotherapy and radiotherapy.

## 2 Materials and methods

### 2.1 Patients

The study included 202 patients treated for various forms of GCTs. The mean age was 46 years with a standard deviation of ± 8.9 years (range 28–69 years). Patients under active surveillance accounted for 15.84% of the sample. Patients managed with active surveillance were followed through a structured monitoring protocol that included regular physical examinations, serum tumor marker assessments, and imaging at predefined intervals, with curative treatment initiated only upon evidence of disease relapse or progression, Radiotherapy consisted of 7.92% of the sample, chemotherapy 69.80%, and chemotherapy+radiotherapy 6.44%. A summary is presented in **Table 1**.

**Table 1:** Patient characteristics summary.

Clinical parameters	All 100%	Treatment groups				
		AS	RT	CT	CTRT	p-value
	N=202	32	16	141	13	
Age (years)	**28-69**					
Median (range)	**46 years** **(28-69)**					
Follow-up (years)	**0-35**					
Median (range)	**13 years** **(0-35)**					
Histology						
Pure seminoma	**67**	**17**	**16**	**24**	**10**	**p = 0,0000**
Non-seminoma/mixed GCT	**129**	**15**	**0**	**111**	**3**	
Histology unknown	**6**	**0**	**0**	**6**	**0**	
Primary tumor						
Gonadal	**186**	**32**	**16**	**126**	**12**	**p = 0,4332**
Primary retroperitoneal	**12**	**0**	**0**	**11**	**0**	
Primary mediastinal	**4**	**0**	**0**	**4**	**0**	
IGCCCG risk group	N= 136					p = 0,0000
Good risk	**90**	**2**	**2**	**78**	**8**	**p = 0,0000**
Intermediate risk	**20**	**0**	**0**	**20**	**0**	
Poor risk	**27**	**0**	**0**	**26**	**1**	
Uncategorized	**65**	**30**	**14**	**17**	**4**	
Initial stage						
I	**69**	**31**	**16**	**16**	**6**	**p = 0,0000**
I.S-III.A	**79**	**1**	**0**	**73**	**5**	
III.B	**24**	**0**	**0**	**23**	**1**	
III.C	**28**	**0**	**0**	**27**	**1**	
Stage unknown	**2**	**0**	**0**	**2**	**0**	
Treatment						
AS	**32**					
RT only	**16**					
• adjuvant RT						
CT only	**141**					
• 1st line only	**124**					
• more than 1st line	**17**					
CTRT	**13**					
• 1st line treatment	**10**					
• 2nd line treatment	**3**					
Initial chemotherapy	**N= 153**					
BEPx3	**54**					
EPx4	**21**					
BEPx4	**45**					
4xVIP	**1**					
4xT-BEP	**6**					
adj.2xBEP	**7**					
other*	**19**					
Cumulative dose of cisplatin						
0 mg/m2	**47**	**31**	**16**	**0**	**0**	**p = 0,0000**
<400 mg/m2	**58**	**1**	**0**	**56**	**1**	
>400 mg/m2	**97**	**0**	**0**	**85**	**12**	
Time from the end of treatment (years)						
0-10	**54**	**19**	**2**	**31**	**2**	**p = 0,0000**
11-15	**82**	**13**	**6**	**59**	**4**	
> 15	**66**	**0**	**8**	**51**	**7**	

*1,6VIP, 3xEP; BEP and taxol-BEP; 4xPVB; 6xCBDCA+CFA + 4xVIP after relapse; 6xTBEP; 2xBEP, 2xEP; 1x CBDCA + 4x VIP; 1xBEP, 3xEP, 4xTIP; 4x HD VIP; VIPx4; 2xCBDCA, 2xBEP;

IGCCCG, International Germ Cell Cancer Collaborative Group; AS, Active surveillance; RT, Radiotherapy; CT, Chemotherapy; CTRT, Chemotherapy and radiotherapy. *p-value ≤ 0,05 was considered statistically significant.

The study included patients treated at the National Cancer Institute in Bratislava from 1988 to 2022. The patients underwent annual checkups as part of long-term follow-up, as stated by the National Cancer Institute (NCI) internal guidelines for the testicular cancer survivorship program. The Ethics Committee of the NCI approved the study, and the patients provided their consent in writing.

Data collected from all participants included age, tumor histology, disease characteristics, dates of orchiectomy, and details of treatment administered. NCI physicians collected the data in the outpatient clinic and entered it into the patient database, which was subsequently extracted for this study. For phenotyping purposes, plasma EDTA (ethylenediaminetetraacetic acid) samples of 3–4 ml and whole blood samples of 1 ml were collected from survivors and examined by flow cytometry during the years 2018-2020. The sample was taken during a follow-up visit and examined the same day.

### 2.2 Study subgroups

We evaluated 27 immune cell types and the resulting immunoregulatory index. Cell counts were compared between the following subgroups of patients according to the treatment:

Active surveillance vs. those administered chemotherapy, active surveillance vs. radiotherapy, and active surveillance vs. combined chemotherapy+radiotherapy. Next, we assessed immune phenotypes between survivors treated with different cumulative doses of cisplatin: 0 mg/m2 vs. ≤ 400 mg/m2, 0 mg/m2 vs. ≥ 400 mg/m2, and ≤ 400 mg/m2 vs. ≥ 400 mg/m2.

### 2.3 Flow-cytometry

For the analysis of cell subpopulations, we used peripheral blood (PB) samples with EDTA processed within 24 hours. For each sample, we incubated the PB sample with monoclonal antibodies conjugated with different fluorochromes for 20 minutes. Antibody clones, dilutions, and target cell populations are listed in **Supplementary Table 2**. We added 2 ml of BD FACS Lyse solution for erythrocyte lysis and incubated again for 15 minutes. Test tubes were then centrifuged for 5 minutes at 1500 rpm. The supernatant was poured off, and the pellet resuspended in 0.5 ml of PBS. The prepared sample was measured on a FACSCanto II flow cytometer equipped with 3 lasers and analyzed with Kaluza analysis software.

We used the following combinations of monoclonal antibodies in three tubes:

1. CD16 FITC (BDB - BD Biosciences) CD56 PE (Sysmex) CD45 PerCP (BDB) CD19 PeCy7 (Beckman Coulter) CD3 APC (BDB) CD8 APCH7 (BDB) CD4 HV450 (BDB) and CD14 HV500 (BDB)
2. CD3 FITC (BDB) CD127 PE (BDB) CD25 PeCy7 (BDP - BD Pharmingen) CD4 HV450 (BDB) and CD45 HV500 (BDB)
3. Lin Coctail2 FITC (BDB) CD1c PE (BDP) CD123 PerCP Cy5.5 (BDP) CD11c APC (BDP) CD16 APCH7 (BDP) HLA DR HV450 (BDB) and CD45 HV500 (BDB)
4. Panel validation was performed on study participant samples; no healthy donor controls were used. For target population analysis, we used the CD45/SSC gating strategy and gating on features typical for the given population: T lymphocytes (CD45^+^CD3^+^CD19^−^CD56^−^CD16^−^), B lymphocytes (CD45^+^CD19^+^), NK cells (CD45^+^CD3^−^CD56^+^CD16^+^/^−^), monocytes (CD45^+^CD14^+^), and dendritic cells (lineage^−^CD45^+^HLA-DR^+^ with CD1c, CD11c, or CD123 expression). A representative gating strategy is shown in **Supplementary Figure 1**.

### 2.4 Statistical analysis

Patient characteristics were categorized in an Excel file and statistically analyzed using the NCSS statistical system (2023).

Data were evaluated using one-way ANOVA, allowing for comprehensive comparisons between multiple groups. In the absence of specific distribution within groups, we used the Kruskal-Wallis test (15). For individual data, we obtained the p-value (considered significant at p = ≤ 0.05), mean, median, and standard deviation.

## 3 Results

### 3.1 Comparison of active surveillance and chemotherapy groups

**Figure 1:**
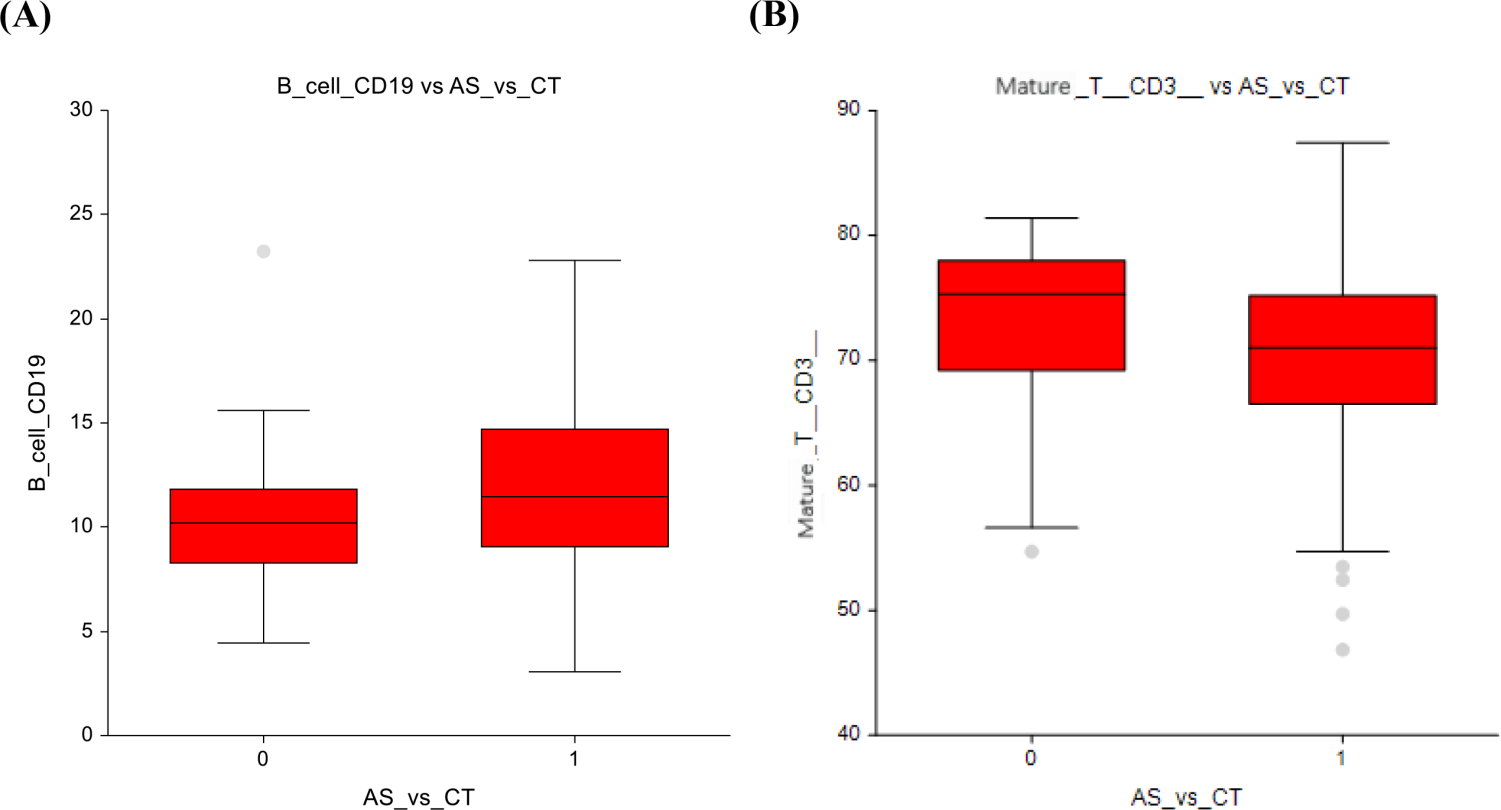
Active surveillance vs. chemotherapy **(A)** B-lymphocytes **(B)** Mature T- lymphocytes.

In our analysis, the AS group exhibited a trend towards lower number of B-lymphocytes (CD19+) compared to the chemotherapy group (10.3 ± 3.5 vs 11.9 ± 4.2; p= 0.04) (**Figure 1A**). Mature T-cells (CD3+) are higher in the AS vs. CT group - in spite of this finding being statistically insignificant, it can still represent a potential clinical relevance, as mature T-cells are essential for cell-mediated immunity (72.9 ± 7.3 vs 70.9 ± 7.5; p=0.07) (**Figure 1B**). The full dataset of this comparison can be found in **Supplementary Table 3**.

### 3.2 Comparison of active surveillance and radiotherapy groups

**Figure 2:**
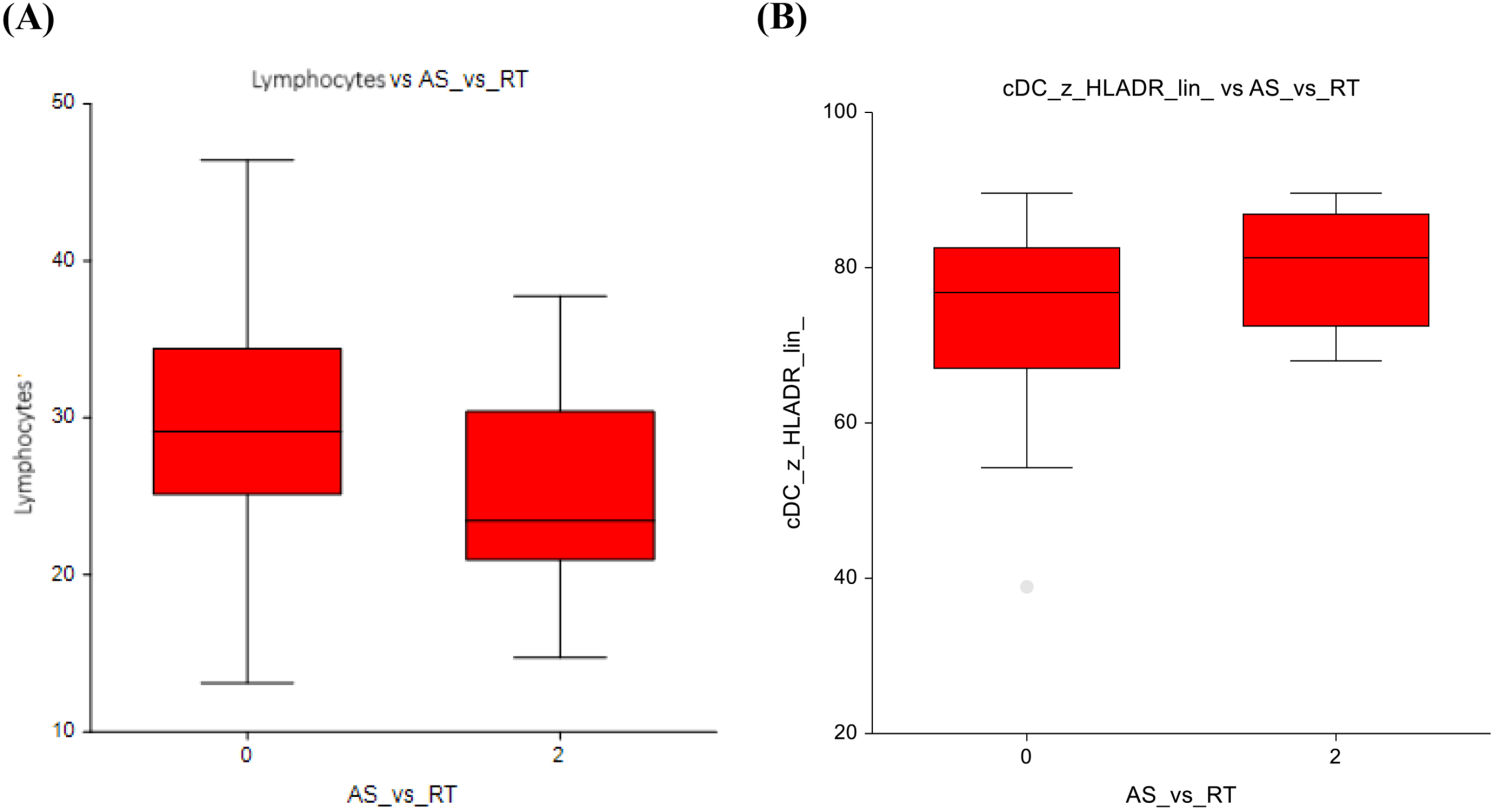
Active surveillance vs. radiotherapy **(A)** Lymphocytes **(B)** Conventional dendritic cells.

The active surveillance group (AS) versus radiotherapy only (RT) group exhibited a higher number of all lymphocytes (29.8 ± 7.7 vs 25.2 ± 6.3; p=0.04) (**Figure 2A**) and a nonsignificant decrease in the number of conventional dendritic cells (74.3 ± 11.8 vs 80.2 ± 7.4; p=0.08) (**Figure 2B**). The full dataset of this comparison can be found in **Supplementary Table 4**.

### 3.3 Comparison of active surveillance and radiotherapy+chemotherapy groups

**Figure 3:**
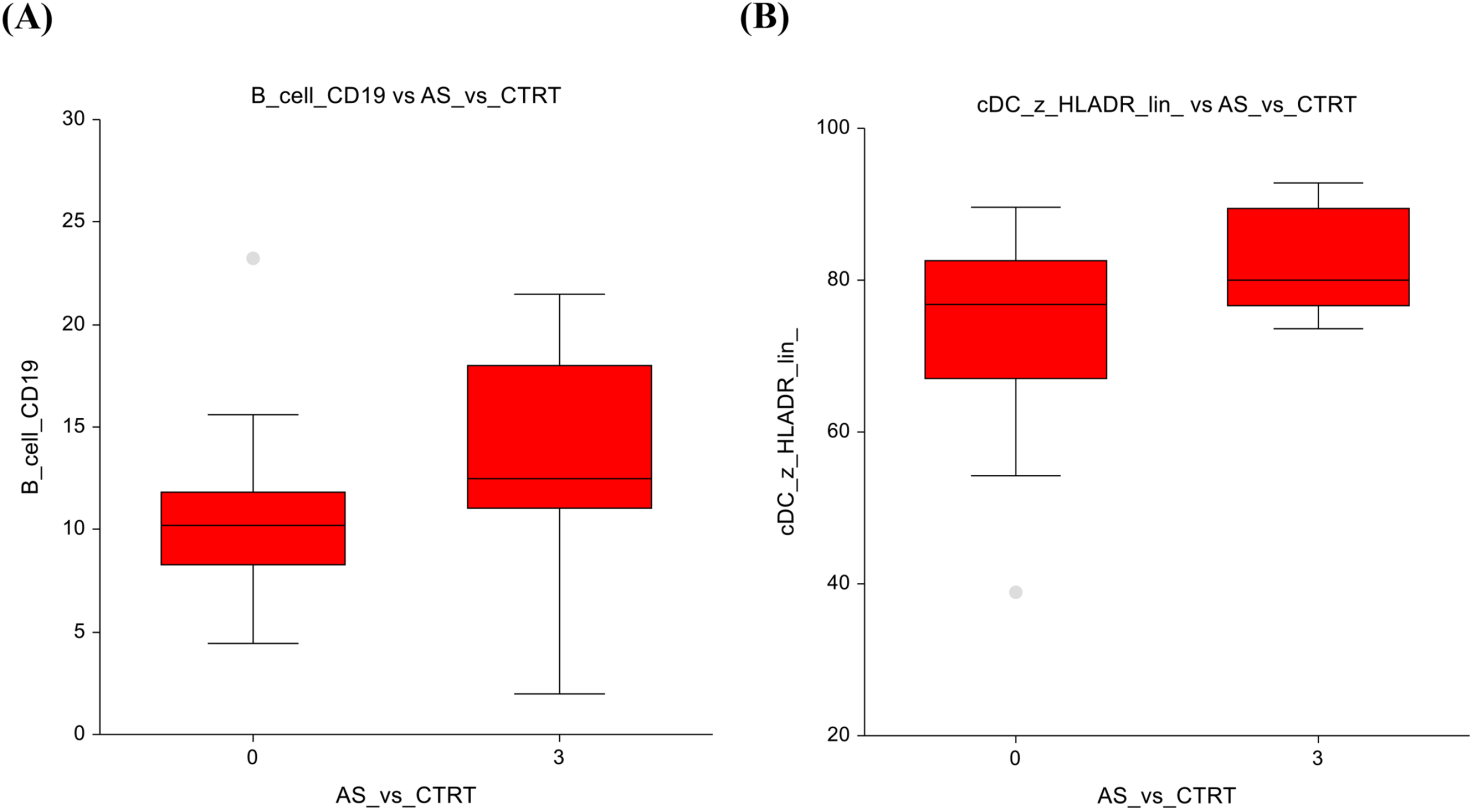
Active surveillance vs. Chemotherapy + radiotherapy **(A)** B-lymphocytes **(B)** Conventional dendritic cells.

When comparing the AS group to the CTRT (chemotherapy+radiotherapy) group, there are notable differences in cell subtypes. Similar to the observations in the AS vs. chemotherapy-only group, the AS vs. CTRT group shows significantly lower number of B-cells (10.3 ± 3.5 vs 13.7 ± 5; p=0.01) (**Figure 3A**) Furthermore, there was a significantly lower number of conventional dendritic cells (74.3 ± 11.8 vs 82.5 ± 6.7; p=0.04) (**Figure 3B**). The full dataset of this comparison can be found in **Supplementary Table 5**.

### 3.4 Comparison of the group without chemotherapy and when administered ≤ 400 mg/m2 of cisplatin-based chemotherapy

**Figure 4:**
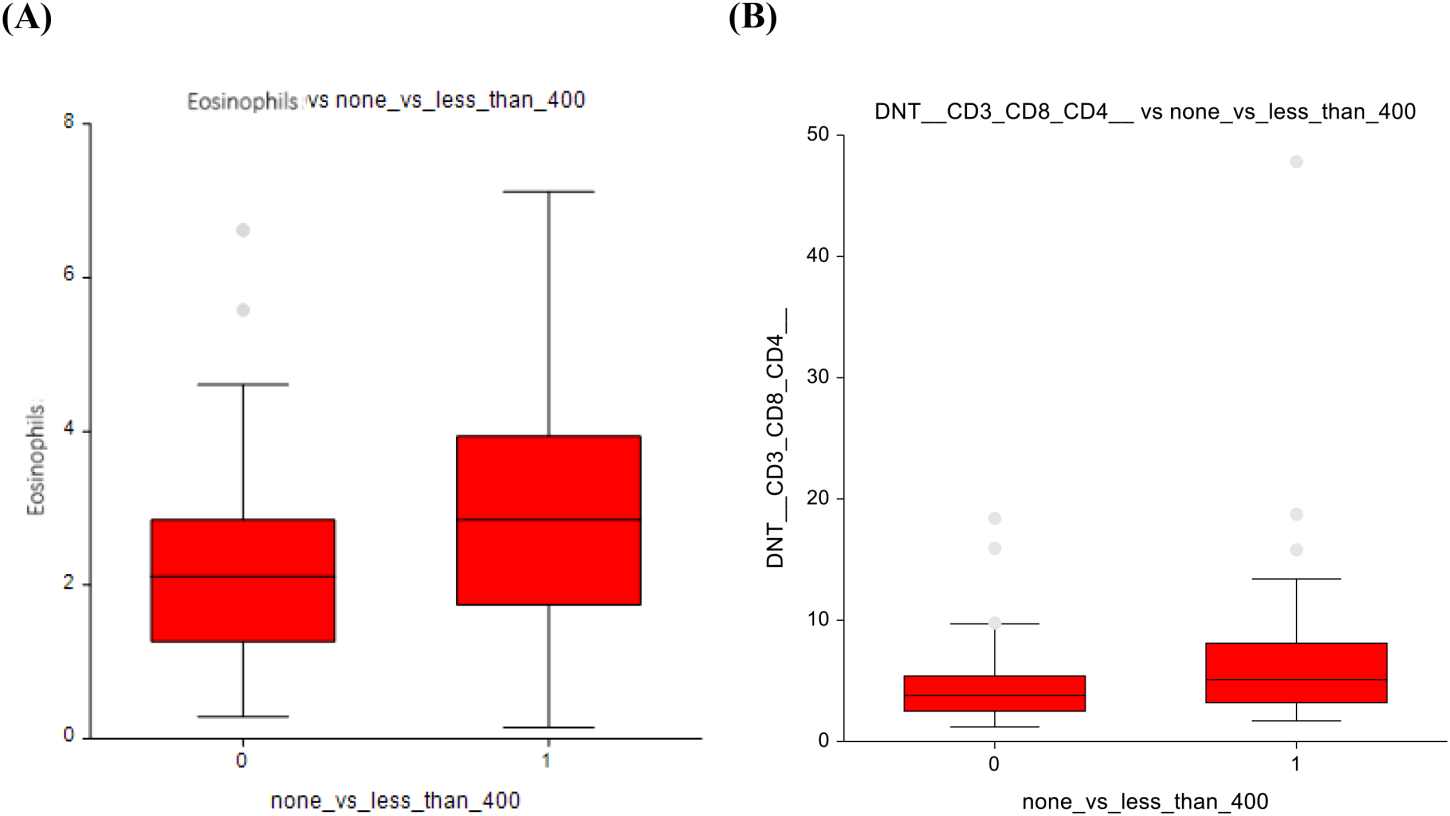
No chemotherapy vs lower-dose chemotherapy **(A)** Eosinophils **(B)** Double-negative T-cells.

When comparing the AS group (0 mg/m²) with those administered ≤ 400 mg/m², several changes in immune cell populations were observed. Eosinophils showed higher numbers (2.3 ± 1.5 vs 2.9 ± 1.7; p=0.03) in the ≤ 400 mg/m² group, suggesting a potential immune response triggered by low-dose chemotherapy (**Figure 4A**). The AS vs lower-dose CT presented significantly lower number of double-negative T-lymphocytes, with a p-value of p=0.03, 4.7 ± 3.4 vs 6.6 ± 6.6 (**Figure 4B**).

**Figure 5:**
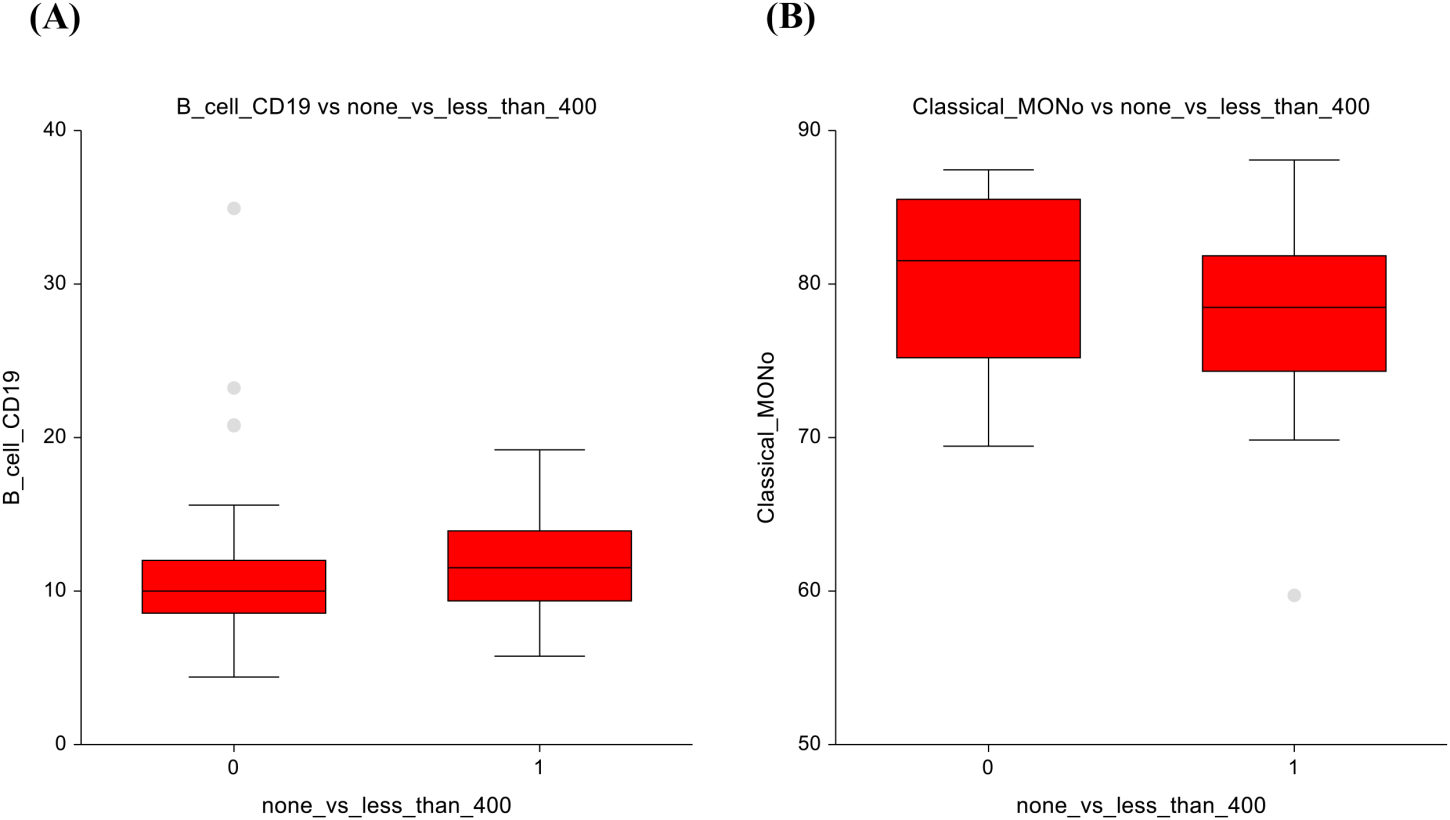
No chemotherapy vs lower-dose chemotherapy results **(A)** B-lymphocytes **(B)** Classical monocytes.

In terms of non-significant changes, B-lymphocytes exhibited a trend towards lower numbers in the AS group and higher numbers in the lower-dose CT group (10.9 ± 5.3 vs 11.6 ± 3.5; p=0.075), suggesting a subtle enhancement of the humoral immune response (**Figure 5A**). Similarly, classical monocytes displayed slightly lower counts under low-dose chemotherapy (80.4± 5.3 vs 78.1 ± 5.4; p = 0.06), suggesting possible modulation of innate immune cell activity **Figure 5B**. The full dataset of this comparison can be found in **Supplementary Table 6**.

### 3.5 Comparison of the group without chemotherapy and with administration of ≥ 400 mg/m2

**Figure 6:**
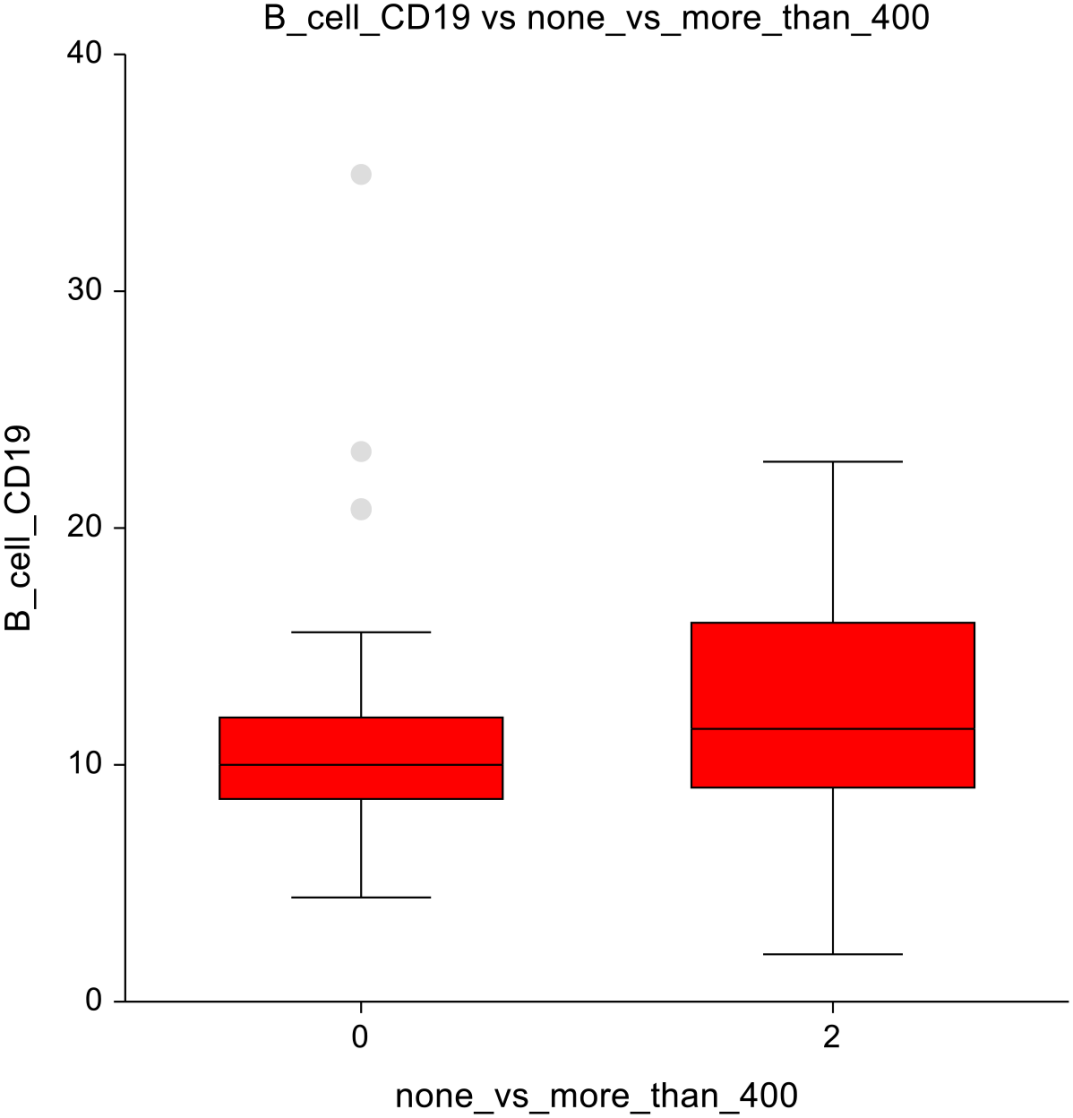
No chemotherapy vs higher-dose chemotherapy - B lymphocytes.

When comparing the AS cohort (0 mg/m²) with doses ≥ 400 mg/m², a notable observation was seen within B-cells, which were decreased with a p-value of 0.03, 10.9 ± 5.3 vs 12.3 ± 4.8 (**Figure 6**). The full dataset of this comparison can be found in **Supplementary Table 7**.

### 3.6 Comparison of the group at administration of ≤ 400 mg/m2 and ≥ 400 mg/m2

**Figure 7:**
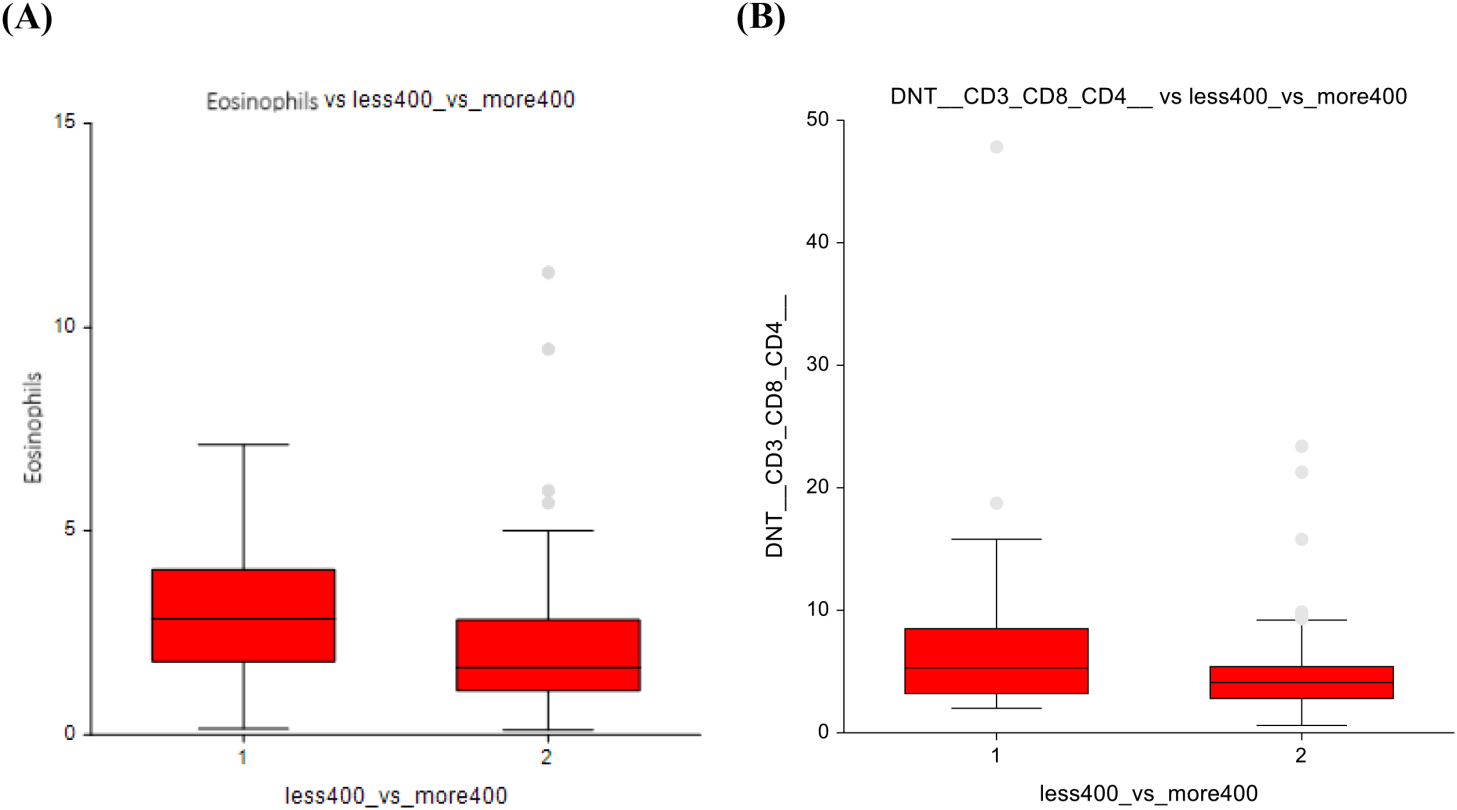
Lower-dose chemotherapy vs higher-dose chemotherapy **(A)** Eosinophils **(B)** Double-negative T-lymphocytes.

**Figure 8:**
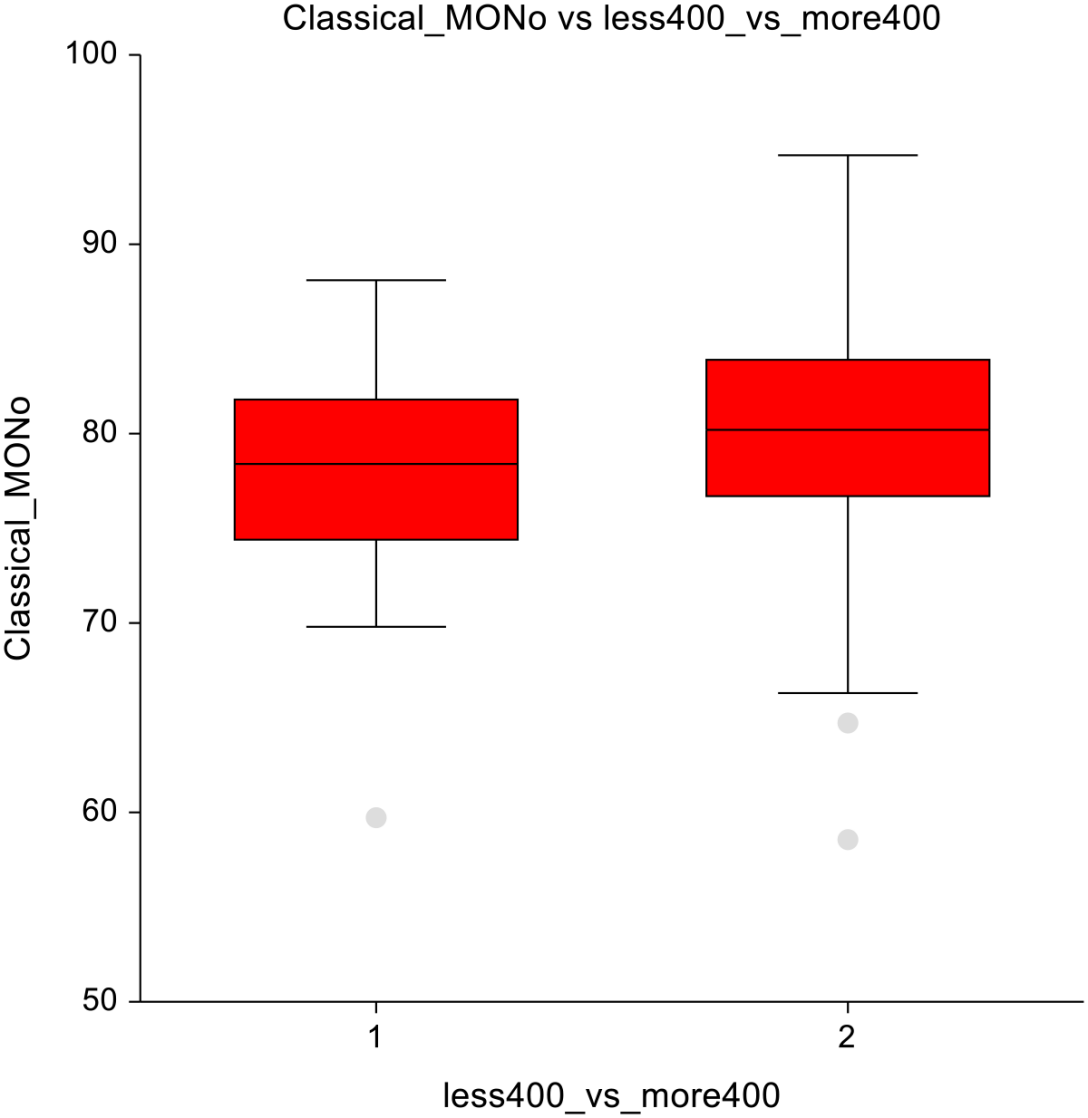
Lower-dose chemotherapy vs higher-dose chemotherapy - classical monocytes.

In lower-dose vs. higher-dose CT group, significant reductions were observed in the higher-dose group. Mainly in eosinophils (3 ± 1.7 vs 2.4 ± 1.7; p = 0.00025) and DNT lymphocytes (6.8 ± 6.7 vs 4.9 ± 3.6; p = 0.02) (**Figures 7A, B**), suggesting suppression of these immune cell subsets at higher chemotherapy dosages. Classical monocytes showed lower numbers in the ≤ 400 mg/m2 group and higher numbers in the ≥ 400 mg/m² group (78 ± 5.4 vs 79.9± 5.9; p=0.07), indicating a potential shift in innate immune cell dynamics in response to higher-dose chemotherapy (**Figure 8**). The full dataset of this comparison can be found in **Supplementary Table 8**.

## 4 Discussion

The data in this study suggests a hypothesis of possible modulatory effects of chemotherapy (CT),radiotherapy (RT) or their combination (CTRT) on the immune profile of long-term germ cell tumor (GCT) survivors, as they represent a population suitable for studying various late treatment sequelae due to high disease curability and survival of many years without recurrence, with the possibility of long-term follow-up. To our knowledge, this is the first study evaluating immune-cell profile in long-term survivors of GCTs.

An interesting point of view is the study of testicular tissue samples and their immune cell and cytokine characteristics, written by Klein et al. Their investigation associated the role of immune cells in the creation of a pro-inflammatory environment with GCTs, especially B-cells, their chemoattractant CXCL-13, and IL-6. When they compared hypospermatogonial samples with germ cell neoplasia *in situ* and seminoma samples, the latter had a significantly higher number of B-cells. (p ≤ 0.006) (16). Although the presence of B-cells does not directly imply an increased humoral immune response, this observation is relevant because pro-inflammatory environments in other tumors are known to contribute to cancer progression (17).

Ziebart et al. looked at the effect of chemotherapy on peripheral blood B-cells in patients with head and neck cancer, with the result of a reduction in the absolute number of CD19+ B-lymphocytes up until a year after cisplatin therapy (18). They were also interested in longer post-therapy intervals in their study and took a blood sample from patients after 14.2 ± 7 months, with the result of an increase in B-lymphocyte frequency and its strong correlation to time post-therapy (p < 0.001). The survivors who received CT or CT+RT in our study also showed an increase in B-lymphocytes. It is unclear how the long-term effects of chemotherapy on immune cell counts are mediated. We may assume chemotherapy and/or radiation may produce long-term activation of inflammatory pathways; however, this is an understudied field.

One of our hypotheses assumes the role of intestinal injury during treatment with subsequent chronic stimulation of the innate immune system due to bacterial translocation from the intestine to the bloodstream. Dysbiosis has been studied and linked to cognitive dysfunction, where gram-negative bacteria release endotoxin lipopolysaccharide (LPS) into the bloodstream, and leads to neuroinflammation by binding to microglial toll-like receptors - this suggests activation of inflammatory cascades and various proinflammatory cytokines (19). In a study by Chovanec et al., biomarkers of gut microbial translocation and dysbiosis were measured from peripheral blood. One of these markers (sCD14), a marker of monocytic activation by lipopolysaccharide, was associated with cognitive impairment in GCT survivors in a long-term follow-up setting. The higher the plasma levels of sCD14, the greater the impairment, especially cognitive impairment perceived by others, but also the overall cognitive function score. Elevated levels of sCD14 point towards ongoing pro-inflammatory signaling in GCT survivors (20).

Another possible explanation for the immune alterations observed in our cohort is the long-term retention of platinum compounds. Previous studies have demonstrated that platinum can remain detectable in serum and tissue for more than 20 years after cisplatin-based chemotherapy (21). We assume that residual levels of cisplatin in serum and tissue may contribute to chronic low-grade inflammation and immune cell modulation, even long after treatment completion. This prolonged presence could partially explain the enduring differences in immune profiles among survivors exposed to cisplatin-containing regimens.

A difference was also observed in a group of conventional dendritic cells (cDCs), which play a key role in antigen presentation and T-cell proliferation, survival and effector function. cDCs can enhance radiotherapy-induced T-cell responses by presenting tumor antigens and stimulating cytotoxic CD8+ T cells, which release cytokines that amplify anti-tumor immunity (22). Platinum-based chemotherapy decreases PDL2 expression by dendritic cells and tumor cells, which drives T-cell responses toward differentiation into Th1 cells and increases the number of tumor antigen-specific T-cells (23). Therefore, the observed increase in cDCs among CT+RT survivors may reflect the combined and lasting effects of these therapies.

No studies address dose-group changes, but our findings suggest a dose-dependent immune effect. We observed cell count elevations in eosinophils and double-negative (DNT) cells with lower doses of chemotherapy (≤ 400 mg/m2), while higher doses led to their reduction. The DNT cells are T lymphocytes that express the T-cell receptor (TCR) but lack CD4 and CD8 co-receptors. Although naturally low in peripheral blood, they play an important role in the immunopathogenesis of autoimmune diseases, viral infections, or tumors. Three or more chemotherapy cycles may decrease these cells, potentially impairing such roles. In cancer, DNT cells appear to play a dual role—either promoting immune suppression within the tumor microenvironment or contributing to anti-tumor immunity, depending on their activation and cytokine profile (24). The dose-dependent differences in DNT cell frequencies observed in our study could therefore reflect long-term immune remodeling after treatment, potentially influencing the balance between immune regulation and surveillance. This dual role proposes an interesting area of future research. Eosinophils, despite being relatively short-lived, also display phenotypic and functional plasticity depending on environmental factors, including the tumor microenvironment, as they can exhibit both anti-tumorigenic and pro-tumorigenic activities, which, as our findings suggest, may be influenced by cumulative chemotherapy dose (25).

While many studies focus primarily on T-cells, particularly cytotoxic T-lymphocytes, we observed no significant variations in our cohort, likely due to the late follow-up (>5 years) when T-cell counts have returned to baseline. For example, this has been observed in patients receiving cisplatin-based chemotherapy with low-dose cyclophosphamide for a variety of malignancies 3 months post-treatment (26). This highlights that long-term immune changes may occur in less-studied cell types, offering novel insights into the interplay between therapy, immune modulation, and late toxicity.

Key strengths of our study are the well-annotated cohort of GCT survivors regularly attending check-ups with decades-long records of their health status, and detailed treatment data to assess the relationship to immune profile.

The limitations involve low numbers of survivors in certain groups, the possible laboratory error in processing blood samples, and incomplete cell-type data. We used active surveillance as our control group instead of healthy cohort, as we consider that the shared exposure to the underlying tumor disease and the uniform surgical intervention (orchiectomy) result in a relatively homogeneous patient population, thereby facilitating the assessment of treatment effects.

Future research is needed to investigate the underlying mechanisms of late toxicity of the therapy, its immunomodulatory capacity, and to refine treatment strategies to mitigate harmful effects or reveal potential therapeutic benefits.

## 5 Conclusion

Our study demonstrates an association between both cisplatin-based chemotherapy and radiotherapy with certain immune cell types, suggesting the immunomodulatory impact of these modalities. We observed both increases and decreases in cell counts; however, additional research is required to better understand both the molecular mechanisms and the clinical implications, as our study is unable to state the results are definitive.

Our comparisons across different treatment groups revealed significant findings. Chemotherapy significantly increased B-lymphocytes (CD19), radiotherapy showed a significant decrease in lymphocytes and a trend towards increased conventional dendritic cells, suggesting a nuanced modulation of immune responses. The combination of radiotherapy and chemotherapy resulted in a significant elevation of B-lymphocytes and conventional dendritic cells. Low-dose chemotherapy (< 400 mg/m²) prompted significant increases in eosinophils and DNT-lymphocytes, while higher doses (> 400 mg/m²) notably reduced eosinophils and DNT-lymphocytes, suggesting dose-dependent effects on immune cell dynamics.

It is imperative to understand the long-term impact of curative cancer treatments on human physiology, to reduce unnecessary overtreatment, and ensure the best quality of life for our patients. Discovering the causality between treatment and immune changes is an important step toward optimizing cancer care.

## Data Availability

The original contributions presented in the study are included in the article/**Supplementary Material**. Further inquiries can be directed to the corresponding author.
