# Supplementary material for: Immune cell profiling of peripheral blood in long-term testicular germ cell tumor survivors
Source: Front Immunol. 2025 Nov 13;16:1697087. doi: 10.3389/fimmu.2025.1697087 (PMC12657365; doi:10.3389/fimmu.2025.1697087)
Supplement: Supplementary file 1 [file Table1.docx]

Supplementary Material

# Supplementary Tables

**Supplementary Table 2:** **Antibody panels used for flow cytometry**

| **Panel** | **Marker** | **Fluorochrome** | **Clone** | **Manufacturer** | **Dilution** | **Target Cell Type** |
| --- | --- | --- | --- | --- | --- | --- |
| Basic | CD16 | FITC | 3G8 | BD Pharmingen | 1:50 | NK cells, monocytes |
| Basic | CD56 | PE | NCAM16.2 | Sysmex | 1:50 | NK cells |
| Basic | CD45 | PerCP | 2D1 | BD Biosciences | 1:100 | All leukocytes |
| Basic | CD19 | PE-Cy7 | SJ25C1 | Beckman Coulter | 1:50 | B cells |
| Basic | CD3 | APC | SK7 | BD Biosciences | 1:50 | T cells |
| Basic | CD8 | APC-H7 | SK1 | BD Biosciences | 1:50 | Cytotoxic T cells |
| Basic | CD4 | V450 | RPA-T4 | BD Biosciences | 1:50 | Helper T cells |
| Basic | CD14 | HV500 | M5E2 | BD Biosciences | 1:50 | Monocytes |
| Regulatory T cell | CD3 | FITC | SK7 | BD Biosciences | 1:50 | T cells |
| Regulatory T cell | CD127 | PE | hIL-7R-M21 | BD Pharmingen | 1:50 | Tregs / activated T cells |
| Regulatory T cell | CD4 | PerCP | SK3 | BD Biosciences | 1:50 | Helper T cells |
| Regulatory T cell | CD25 | PE-Cy7 | 2A3 | BD Biosciences | 1:50 | Tregs |
| Regulatory T cell | CD45 | HV450 | HI30 | BD Biosciences | 1:50 | All leukocytes |
| Dendritic cell | Lin cocktail 2 | FITC | — | BD Biosciences | 1:50 | Lineage-negative cells |
| Dendritic cell | CD1c | PE | F10/21A3 | BD Pharmingen | 1:50 | Myeloid DCs |
| Dendritic cell | CD123 | PerCP-Cy5.5 | 7G3 | BD Pharmingen | 1:50 | Plasmacytoid DCs |
| Dendritic cell | CD11c | APC | B-ly6 | BD Pharmingen | 1:50 | Myeloid DCs |
| Dendritic cell | CD16 | APC-H7 | 3G8 | BD Pharmingen | 1:50 | NK cells, monocytes |
| Dendritic cell | HLA-DR | HV450 | L243 | BD Biosciences | 1:50 | Antigen-presenting cells |
| Dendritic cell | CD45 | HV500 | HI30 | BD Biosciences | 1:50 | All leukocytes |

**Supplementary Table 3: Comparison of active surveillance and chemotherapy groups**

| Neutrophils | n | mean | median | SD | p-value |
| --- | --- | --- | --- | --- | --- |
| AS | 32 | 59,26 | 60,01 | 8,47 | 0,83890 |
| CT | 141 | 59,85 | 59,88 | 8,47 |  |
| Lymphocytes | n | mean | median | SD | p-value |
| AS | 32 | 29,81 | 29,145 | 7,67 | 0,98596 |
| CT | 141 | 29,38 | 29,37 | 7,796 |  |
| Monocytes | n | mean | median | SD | p-value |
| AS | 32 | 8,78 | 9,025 | 1,75 | 0,82822 |
| CT | 141 | 8,97 | 8,77 | 2,25 |  |
| Eosinophils | n | mean | median | SD | p-value |
| AS | 32 | 2,29 | 1,88 | 1,67 | 0,54581 |
| CT | 141 | 2,48 | 2,15 | 1,73 |  |
| Basophils | n | mean | median | SD | p-value |
| AS | 32 | 0,83 | 0,765 | 0,458 | 0,11781 |
| CT | 141 | 0,72 | 0,62 | 0,482 |  |
| B-cells (CD19) | n | mean | median | SD | p-value |
| AS | 32 | 10,31 | 10,22 | 3,53 | **0,04050** |
| CT | 141 | 11,87 | 11,48 | 4,23 |  |
| Mature T-cells (CD3+) | n | mean | median | SD | p-value |
| AS | 32 | 72,93 | 75,335 | 7,27 | 0,07180 |
| CT | 141 | 70,895 | 71,02 | 7,51 |  |
| Helper T-lymphocytes | n | mean | median | SD | p-value |
| AS | 32 | 43,41 | 42,535 | 6,76 | 0,42854 |
| CT | 141 | 41,61 | 42,25 | 8,36 |  |
| Cytotoxic T-lymphocytes | n | mean | median | SD | p-value |
| AS | 32 | 24,997 | 25,42 | 7,21 | 0,33323 |
| CT | 141 | 23,8338 | 22,82 | 7,50 |  |
| NKT cells | n | mean | median | SD | p-value |
| AS | 32 | 4,39 | 3,79 | 2,98 | 0,90973 |
| CT | 141 | 4,97 | 3,6 | 4,69 |  |
| NK cells | n | mean | median | SD | p-value |
| AS | 32 | 14,797 | 13,11 | 7,14 | 0,59358 |
| CT | 141 | 15,42 | 13,94 | 7,06 |  |
| Regulatory T -cells | n | mean | median | SD | p-value |
| AS | 32 | 7,966 | 8,325 | 1,90 | 0,68093 |
| CT | 140 | 8,22 | 8,005 | 2,13 |  |
| Classic monocytes | n | mean | median | SD | p-value |
| AS | 27 | 79,707 | 80,24 | 5,37 | 0,73761 |
| CT | 126 | 79,299 | 79,85 | 5,694 |  |
| Intermediate monocytes | n | mean | median | SD | p-value |
| AS | 27 | 4,49 | 3,53 | 2,71 | 0,72661 |
| CT | 124 | 4,42 | 4,035 | 2,2898 |  |
| Nonclassical monocytes | n | mean | median | SD | p-value |
| AS | 27 | 9,63 | 7,35 | 4,51 | 0,37419 |
| CT | 124 | 10,08 | 9,1 | 4,18 |  |
| PMN -MDSC | n | mean | median | SD | p-value |
| AS | 14 | 0,575 | 0,305 | 0,62 | 0,97541 |
| CT | 68 | 0,518 | 0,33 | 0,64 |  |
| M-MDSC ( % total monocytes) | n | mean | median | SD | p-value |
| AS | 14 | 5,96 | 5,285 | 3,596 | 0,82925 |
| CT | 68 | 5,88 | 5,41 | 2,78 |  |
| PMN ( % total leukocytes) | n | mean | median | SD | p-value |
| AS | 14 | 0,17 | 0,065 | 0,28 | 0,89200 |
| CT | 68 | 0,11 | 0,08 | 0,13 |  |
| M-MDSC ( % total leukocytes) | n | mean | median | SD | p-value |
| AS | 14 | 3,965 | 3,465 | 2,595 | 0,78629 |
| CT | 68 | 3,93 | 3,875 | 2,12 |  |
| Conventional DC HLADR+ lin- | n | mean | median | SD | p-value |
| AS | 30 | 74,33 | 76,775 | 11,75 | 0,11246 |
| CT | 127 | 78,05 | 79,43 | 9,57 |  |
| Plasmacytoid DC HLADR+ lin- | n | mean | median | SD | p-value |
| AS | 20 | 12,61 | 11,6 | 5,89 | 0,51465 |
| CT | 97 | 11,56 | 11,04 | 5,14 |  |
| cDC ( % total leukocytes) | n | mean | median | SD | p-value |
| AS | 30 | 1,047 | 0,9 | 0,50 | 0,33593 |
| CT | 127 | 1,079 | 1,01 | 0,41 |  |
| pDC ( % total leukocytes) | n | mean | median | SD | p-value |
| AS | 20 | 0,149 | 0,15 | 0,05 | 0,68735 |
| CT | 98 | 0,15 | 0,14 | 0,07 |  |
| CD16+ DC  lin- HLADR+ | n | mean | median | SD | p-value |
| AS | 18 | 53,15 | 57,375 | 16,99 | 0,16363 |
| CT | 67 | 59,14 | 60,49 | 12,48 |  |
| DC2 (% DC) | n | mean | median | SD | p-value |
| AS | 23 | 17,26 | 17,36 | 6,65 | 0,32009 |
| CT | 111 | 16,73 | 14,97 | 8,09 |  |
| Immunoregulatory index (CD4+/CD8+) | n | mean | median | SD | p-value |
| AS | 31 | 1,96 | 1,79 | 0,8 | 0,97934 |
| CT | 141 | 2,01 | 1,83 | 1,00 |  |
| DNT- cells (CD3+CD8-CD4-) | n | mean | median | SD | p-value |
| AS | 32 | 4,52 | 3,345 | 3,32 | 0,13077 |
| CT | 141 | 5,44 | 4,22 | 4,97 |  |

**Supplementary Table 4: Comparison of active surveillance and radiotherapy groups**

| Neutrophils | n | mean | median | SD | p-value |
| --- | --- | --- | --- | --- | --- |
| AS | 32 | 59,26 | 60,01 | 8,47 | 0,11533 |
| RT | 16 | 63,04 | 63,705 | 7,17 |  |
| Lymphocytes | n | mean | median | SD | p-value |
| AS | 32 | 29,81 | 29,145 | 7,69 | **0,04195** |
| RT | 16 | 25,23 | 23,425 | 6,33 |  |
| Monocytes | n | mean | median | SD | p-value |
| AS | 32 | 8,78 | 9,025 | 1,75 | 0,09868 |
| RT | 16 | 10,035 | 10,16 | 2,75 |  |
| Eosinophils | n | mean | median | SD | p-value |
| AS | 32 | 2,29 | 1,88 | 1,67 | 0,46371 |
| RT | 16 | 2,24 | 2,12 | 0,905 |  |
| Basophils | n | mean | median | SD | p-value |
| AS | 32 | 0,83 | 0,77 | 0,49 | 0,08398 |
| RT | 16 | 0,59 | 0,6 | 0,25 |  |
| B-cells (CD19) | n | mean | median | SD | p-value |
| AS | 32 | 10,31 | 10,22 | 3,53 | 0,93029 |
| RT | 16 | 12,06 | 9,76 | 7,63 |  |
| Mature T-cells (CD3+) | n | mean | median | SD | p-value |
| AS | 32 | 72,93 | 75,335 | 7,27 | 0,22902 |
| RT | 16 | 69,25 | 72,83 | 10,45 |  |
| Helper T-lymphocytes | n | mean | median | SD | p-value |
| AS | 32 | 43,41 | 42,54 | 6,76 | 0,11037 |
| RT | 16 | 38,57 | 38,89 | 7,89 |  |
| Cytotoxic T-lymphocytes | n | mean | median | SD | p-value |
| AS | 32 | 24,997 | 25,42 | 7,205 | 1,00000 |
| RT | 16 | 24,94 | 23,49 | 7,205 |  |
| NKT cells | n | mean | median | SD | p-value |
| AS | 32 | 4,39 | 3,79 | 2,98 | 0,11034 |
| RT | 16 | 7,4 | 4,425 | 5,72 |  |
| NK cells | n | mean | median | SD | p-value |
| AS | 32 | 14,78 | 13,11 | 7,14 | 0,47046 |
| RT | 16 | 16,33 | 14,5 | 8,06 |  |
| Regulatory T -cells | n | mean | median | SD | p-value |
| AS | 32 | 7,97 | 8,325 | 1,9 | 0,16154 |
| RT | 16 | 8,73 | 9,08 | 2,31 |  |
| Classic monocytes | n | mean | median | SD | p-value |
| AS | 27 | 79,7 | 80,24 | 5,37 | 0,34799 |
| RT | 13 | 81,56 | 83,02 | 4,76 |  |
| Intermediate monocytes | n | mean | median | SD | p-value |
| AS | 27 | 4,49 | 3,53 | 2,71 | 0,91950 |
| RT | 13 | 4,36 | 4,38 | 2,18 |  |
| Nonclassical monocytes | n | mean | median | SD | p-value |
| AS | 27 | 9,63 | 7,35 | 4,51 | 0,69665 |
| RT | 13 | 9,097 | 8,39 | 4,27 |  |
| PMN -MDSC | n | mean | median | SD | p-value |
| AS | 14 | 0,575 | 0,305 | 0,62 | 0,43212 |
| RT | 8 | 0,35 | 0,36 | 0,298 |  |
| M-MDSC ( % total monocytes) | n | mean | median | SD | p-value |
| AS | 14 | 5,96 | 5,285 | 3,596 | 0,83776 |
| RT | 8 | 6,165 | 5,125 | 3,05 |  |
| PMN ( % total leukocytes) | n | mean | median | SD | p-value |
| AS | 14 | 0,17 | 0,065 | 0,28 | 0,70674 |
| RT | 8 | 0,08 | 0,065 | 0,07 |  |
| M-MDSC ( % total leukocytes) | n | mean | median | SD | p-value |
| AS | 14 | 3,965 | 3,47 | 2,595 | 0,94558 |
| RT | 8 | 4,03 | 3,69 | 2,35 |  |
| Conventional DC HLADR+ lin- | n | mean | median | SD | p-value |
| AS | 30 | 74,33 | 76,78 | 11,75 | 0,08788 |
| RT | 16 | 80,22 | 81,27 | 7,39 |  |
| Plasmacytoid DC HLADR+ lin- | n | mean | median | SD | p-value |
| AS | 20 | 12,61 | 11,6 | 5,89 | 0,31984 |
| RT | 13 | 10,16 | 8,64 | 4,83 |  |
| cDC ( % total leukocytes) | n | mean | median | SD | p-value |
| AS | 30 | 1,047 | 0,9 | 0,50 | 0,65282 |
| RT | 16 | 1,11 | 1,045 | 0,54 |  |
| pDC ( % total leukocytes) | n | mean | median | SD | p-value |
| AS | 20 | 0,15 | 0,15 | 0,05 | 0,36509 |
| RT | 13 | 0,13 | 0,12 | 0,06 |  |
| CD16+ DC  lin- HLADR+ | n | mean | median | SD | p-value |
| AS | 18 | 53,15 | 57,38 | 16,99 | 0,34523 |
| RT | 11 | 61,18 | 55,95 | 10,57 |  |
| DC2 (% DC) | n | mean | median | SD | p-value |
| AS | 23 | 17,26 | 17,36 | 6,65 | 0,26623 |
| RT | 14 | 15,07 | 15,46 | 4,78 |  |
| Immunoregulatory index (CD4+/CD8+) | n | mean | median | SD | p-value |
| AS | 31 | 1,96 | 1,79 | 0,81 | 0,36917 |
| RT | 16 | 1,696 | 1,59 | 0,65 |  |
| DNT- cells (CD3+CD8-CD4-) | n | mean | median | SD | p-value |
| AS | 32 | 4,52 | 3,35 | 3,32 | 0,12046 |
| RT | 16 | 5,73 | 5,1 | 3,97 |  |

**Supplementary Table 5: Comparison of active surveillance and radiotherapy + chemotherapy groups**

| Neutrophils | n | mean | median | SD | p-value |
| --- | --- | --- | --- | --- | --- |
| AS | 32 | 59,26 | 60,01 | 8,47 | 0,29868 |
| CTRT | 13 | 62,56 | 64,74 | 11,53 |  |
| Lymphocytes | n | mean | median | SD | p-value |
| AS | 32 | 29,81 | 29,145 | 7,69 | 0,24934 |
| CTRT | 13 | 26,45 | 25,78 | 9,04 |  |
| Monocytes | n | mean | median | SD | p-value |
| AS | 32 | 8,78 | 9,03 | 1,75 | 0,39449 |
| CTRT | 13 | 9,07 | 7,78 | 3,98 |  |
| Eosinophils | n | mean | median | SD | p-value |
| AS | 32 | 2,29 | 1,88 | 1,67 | 0,83141 |
| CTRT | 13 | 2,35 | 1,83 | 2,26 |  |
| Basophils | n | mean | median | SD | p-value |
| AS | 32 | 0,83 | 0,765 | 0,49 | 0,81193 |
| CTRT | 13 | 0,74 | 0,73 | 0,31 |  |
| B-cells (CD19) | n | mean | median | SD | p-value |
| AS | 32 | 10,31 | 10,22 | 3,53 | **0,01317** |
| CTRT | 13 | 13,69 | 12,48 | 5,07 |  |
| Mature T-cells (CD3+) | n | mean | median | SD | p-value |
| AS | 32 | 72,93 | 75,34 | 7,27 | 0,35416 |
| CTRT | 13 | 71,26 | 71,01 | 8,198 |  |
| Helper T-lymphocytes | n | mean | median | SD | p-value |
| AS | 32 | 43,41 | 42,54 | 6,76 | 0,98002 |
| CTRT | 13 | 42,01 | 43,60 | 8,61 |  |
| Cytotoxic T-lymphocytes | n | mean | median | SD | p-value |
| AS | 32 | 24,997 | 25,42 | 7,21 | 0,29290 |
| CTRT | 13 | 23,59 | 22,68 | 11,42 |  |
| NKT cells | n | mean | median | SD | p-value |
| AS | 32 | 4,39 | 3,79 | 2,98 | 0,43019 |
| CTRT | 13 | 5,78 | 4,95 | 5,56 |  |
| NK cells | n | mean | median | SD | p-value |
| AS | 32 | 14,798 | 13,11 | 7,14 | 0,84122 |
| CTRT | 13 | 13,49 | 13,6 | 5,92 |  |
| Regulatory T -cells | n | mean | median | SD | p-value |
| AS | 32 | 7,97 | 8,325 | 1,902 | 0,58895 |
| CTRT | 13 | 8,405 | 8,185 | 2,05 |  |
| Classic monocytes | n | mean | median | SD | p-value |
| AS | 27 | 79,71 | 80,24 | 5,37 | 0,83742 |
| CTRT | 10 | 78,57 | 79,725 | 7,23 |  |
| Intermediate monocytes | n | mean | median | SD | p-value |
| AS | 27 | 4,49 | 3,53 | 2,71 | 0,94574 |
| CTRT | 10 | 4,67 | 4,08 | 2,99 |  |
| Nonclassical monocytes | n | mean | median | SD | p-value |
| AS | 27 | 9,63 | 7,35 | 4,51 | 0,37388 |
| CTRT | 10 | 11,047 | 10,415 | 5,72 |  |
| PMN -MDSC | n | mean | median | SD | p-value |
| AS | 14 | 0,575 | 0,305 | 0,62 | 0,85190 |
| CTRT | 7 | 0,47 | 0,42 | 0,39 |  |
| M-MDSC ( % total monocytes) | n | mean | median | SD | p-value |
| AS | 14 | 5,96 | 5,29 | 3,596 | 0,94053 |
| CTRT | 7 | 6,89 | 4,65 | 5,82 |  |
| PMN ( % total leukocytes) | n | mean | median | SD | p-value |
| AS | 14 | 0,166 | 0,065 | 0,28 | 0,39046 |
| CTRT | 7 | 0,27 | 0,11 | 0,31 |  |
| M-MDSC ( % total leukocytes) | n | mean | median | SD | p-value |
| AS | 14 | 3,965 | 3,465 | 2,595 | 0,82290 |
| CTRT | 7 | 4,95 | 3,64 | 4,21 |  |
| Conventional DC HLADR+ lin- | n | mean | median | SD | p-value |
| AS | 30 | 74,33 | 76,775 | 11,75 | **0,03670** |
| CTRT | 13 | 82,53 | 80,07 | 6,72 |  |
| Plasmacytoid DC HLADR+ lin- | n | mean | median | SD | p-value |
| AS | 20 | 12,61 | 11,6 | 5,89 | 0,18689 |
| CTRT | 10 | 9,375 | 8,73 | 5,31 |  |
| cDC ( % total leukocytes) | n | mean | median | SD | p-value |
| AS | 18 | 1,047 | 0,9 | 0,50 | 0,41226 |
| CTRT | 8 | 1,21 | 1,1 | 0,66 |  |
| pDC ( % total leukocytes) | n | mean | median | SD | p-value |
| AS | 20 | 0,149 | 0,15 | 0,05 | 0,20838 |
| CTRT | 10 | 0,128 | 0,13 | 0,07 |  |
| CD16+ DC  lin- HLADR+ | n | mean | median | SD | p-value |
| AS | 18 | 53,15 | 57,375 | 16,99 | 0,14861 |
| CTRT | 8 | 64,46 | 62,79 | 9,23 |  |
| DC2 (% DC) | n | mean | median | SD | p-value |
| AS | 23 | 17,26 | 17,36 | 6,65 | 0,10930 |
| CTRT | 11 | 13,72 | 15,13 | 5,695 |  |
| Immunoregulatory index (CD4+/CD8+) | n | mean | median | SD | p-value |
| AS | 31 | 1,96 | 1,789 | 0,81 | 0,34778 |
| CTRT | 13 | 2,23 | 2,06 | 1,17 |  |
| DNT- cells (CD3+CD8-CD4-) | n | mean | median | SD | p-value |
| AS | 32 | 4,52 | 3,345 | 3,32 | 0,17628 |
| CTRT | 13 | 5,67 | 4,23 | 4,90 |  |

**Supplementary Table 6: Comparison of the group without chemotherapy and when administered less than 400 mg/m2**

| Neutrophils | n | mean | median | SD | p-value |
| --- | --- | --- | --- | --- | --- |
| 0 mg/m^2^ | 47 | 60,57 | 61,16 | 8,26 | 0,33858 |
| ≤ 400 mg/m^2^ | 58 | 59,26 | 59,415 | 8,41 |  |
| Lymphocytes | n | mean | median | SD | p-value |
| 0 mg/m^2^ | 47 | 28,27 | 26,69 | 7,599 | 0,16296 |
| ≤ 400 mg/m^2^ | 58 | 29,92 | 29,265 | 7,23 |  |
| Monocytes | n | mean | median | SD | p-value |
| 0 mg/m^2^ | 47 | 9,22 | 9,26 | 2,21 | 0,35508 |
| ≤ 400 mg/m^2^ | 58 | 8,91 | 8,72 | 2,16 |  |
| Eosinophils | n | mean | median | SD | p-value |
| 0 mg/m^2^ | 47 | 2,29 | 2,11 | 1,46 | **0,02619** |
| ≤ 400 mg/m^2^ | 58 | 2,99 | 2,84 | 1,72 |  |
| Basophils | n | mean | median | SD | p-value |
| 0 mg/m^2^ | 47 | 0,72 | 0,7 | 0,38 | 0,97943 |
| ≤ 400 mg/m^2^ | 58 | 0,75 | 0,63 | 0,41 |  |
| B-cells (CD19) | n | mean | median | SD | p-value |
| 0 mg/m^2^ | 47 | 10,96 | 9,97 | 5,28 | 0,07583 |
| ≤ 400 mg/m^2^ | 58 | 11,57 | 11,54 | 3,45 |  |
| Mature T-cells (CD3+) | n | mean | median | SD | p-value |
| 0 mg/m^2^ | 47 | 71,62 | 73,7 | 8,60 | 0,56846 |
| ≤ 400 mg/m^2^ | 58 | 72,34 | 72,67 | 5,84 |  |
| Helper T-lymphocytes | n | mean | median | SD | p-value |
| 0 mg/m^2^ | 47 | 41,78 | 42,16 | 7,51 | 0,79161 |
| ≤ 400 mg/m^2^ | 58 | 41,55 | 41,88 | 8,79 |  |
| Cytotoxic T-lymphocytes | n | mean | median | SD | p-value |
| 0 mg/m^2^ | 47 | 25,09 | 24,46 | 7,16 | 0,25402 |
| ≤ 400 mg/m^2^ | 58 | 24,16 | 22,71 | 6,44 |  |
| NKT cells | n | mean | median | SD | p-value |
| 0 mg/m^2^ | 47 | 5,47 | 4,09 | 4,296 | 0,54467 |
| ≤ 400 mg/m^2^ | 58 | 5,08 | 3,92 | 4,40 |  |
| NK cells | n | mean | median | SD | p-value |
| 0 mg/m^2^ | 47 | 15,35 | 13,71 | 7,48 | 0,49826 |
| ≤ 400 mg/m^2^ | 58 | 14,16 | 13,19 | 5,87 |  |
| Regulatory T -cells | n | mean | median | SD | p-value |
| 0 mg/m^2^ | 47 | 8,27 | 8,45 | 2,05 | 0,93067 |
| ≤ 400 mg/m^2^ | 58 | 8,41 | 8,345 | 2,17 |  |
| Classic monocytes | n | mean | median | SD | p-value |
| 0 mg/m^2^ | 39 | 80,35 | 81,52 | 5,25 | 0,05558 |
| ≤ 400 mg/m^2^ | 50 | 78,092 | 78,44 | 5,37 |  |
| Intermediate monocytes | n | mean | median | SD | p-value |
| 0 mg/m^2^ | 39 | 4,43 | 3,53 | 2,55 | 0,82333 |
| ≤ 400 mg/m^2^ | 50 | 4,14 | 3,94 | 2,06 |  |
| Nonclassical monocytes | n | mean | median | SD | p-value |
| 0 mg/m^2^ | 39 | 9,53 | 8,39 | 4,42 | 0,11143 |
| ≤ 400 mg/m^2^ | 50 | 10,61 | 10,48 | 3,989 |  |
| PMN -MDSC | n | mean | median | SD | p-value |
| 0 mg/m^2^ | 22 | 0,49 | 0,335 | 0,53 | 0,53924 |
| ≤ 400 mg/m^2^ | 31 | 0,54 | 0,29 | 0,52 |  |
| M-MDSC ( % total monocytes) | n | mean | median | SD | p-value |
| 0 mg/m^2^ | 22 | 6,03 | 5,18 | 3,33 | 0,94244 |
| ≤ 400 mg/m^2^ | 31 | 5,82 | 5,34 | 3,03 |  |
| PMN ( % total leukocytes) | n | mean | median | SD | p-value |
| 0 mg/m^2^ | 22 | 0,135 | 0,065 | 0,23 | 0,89925 |
| ≤ 400 mg/m^2^ | 31 | 0,13 | 0,08 | 0,17 |  |
| M-MDSC ( % total leukocytes) | n | mean | median | SD | p-value |
| 0 mg/m^2^ | 45 | 3,99 | 3,465 | 2,45 | 0,54536 |
| ≤ 400 mg/m^2^ | 53 | 4,21 | 4,14 | 2,23 |  |
| Conventional DC HLADR+ lin- | n | mean | median | SD | p-value |
| 0 mg/m^2^ | 45 | 76,49 | 80,19 | 10,82 | 0,48033 |
| ≤ 400 mg/m^2^ | 53 | 78,28 | 79,9 | 9,54 |  |
| Plasmacytoid DC HLADR+ lin- | n | mean | median | SD | p-value |
| 0 mg/m^2^ | 33 | 11,65 | 11,06 | 5,55 | 0,82769 |
| ≤ 400 mg/m^2^ | 37 | 11,82 | 11,63 | 5,33 |  |
| cDC ( % total leukocytes) | n | mean | median | SD | p-value |
| 0 mg/m^2^ | 45 | 1,076 | 0,92 | 0,51 | 0,27533 |
| ≤ 400 mg/m^2^ | 53 | 1,13 | 1,08 | 0,41 |  |
| pDC ( % total leukocytes) | n | mean | median | SD | p-value |
| 0 mg/m^2^ | 33 | 0,14 | 0,15 | 0,05 | 0,33170 |
| ≤ 400 mg/m^2^ | 38 | 0,15 | 0,16 | 0,06 |  |
| CD16+ DC  lin- HLADR+ | n | mean | median | SD | p-value |
| 0 mg/m^2^ | 28 | 56,62 | 57,38 | 15,27 | 0,73325 |
| ≤ 400 mg/m^2^ | 32 | 58,59 | 59,66 | 11,16 |  |
| DC2 (% DC) | n | mean | median | SD | p-value |
| 0 mg/m^2^ | 37 | 16,43 | 17,25 | 6,04 | 0,71977 |
| ≤ 400 mg/m^2^ | 45 | 18,56 | 15,32 | 10,52 |  |
| Immunoregulatory index (CD4+/CD8+) | n | mean | median | SD | p-value |
| 0 mg/m^2^ | 46 | 1,87 | 1,72 | 0,77 | 0,63747 |
| ≤ 400 mg/m^2^ | 58 | 1,88 | 1,82 | 0,74 |  |
| DNT- cells (CD3+CD8-CD4-) | n | mean | median | SD | p-value |
| 0 mg/m^2^ | 47 | 4,74 | 3,77 | 3,36 | **0,03318** |
| ≤ 400 mg/m^2^ | 58 | 6,62 | 5,08 | 6,60 |  |

**Supplementary Table 7: Comparison of the group without chemotherapy and with administration of more than 400 mg/m2**

| Neutrophils | n | mean | median | SD | p-value |
| --- | --- | --- | --- | --- | --- |
| 0 mg/m^2^ | 47 | 60,57 | 61,16 | 8,27 | 0,85129 |
| ≥ 400 mg/m^2^ | 97 | 60,55 | 60,45 | 8,84 |  |
| Lymphocytes | n | mean | median | SD | p-value |
| 0 mg/m^2^ | 47 | 28,27 | 26,69 | 7,599 | 0,50763 |
| ≥ 400 mg/m^2^ | 97 | 28,67 | 28,41 | 8,26 |  |
| Monocytes | n | mean | median | SD | p-value |
| 0 mg/m^2^ | 47 | 9,22 | 9,26 | 2,21 | 0,43306 |
| ≥ 400 mg/m^2^ | 97 | 9,01 | 8,71 | 2,57 |  |
| Eosinophils | n | mean | median | SD | p-value |
| 0 mg/m^2^ | 47 | 2,297 | 2,11 | 1,46 | 0,24475 |
| ≥ 400 mg/m^2^ | 97 | 2,14 | 1,66 | 1,73 |  |
| Basophils | n | mean | median | SD | p-value |
| 0 mg/m^2^ | 47 | 0,72 | 0,7 | 0,38 | 0,46622 |
| ≥ 400 mg/m^2^ | 97 | 0,71 | 0,62 | 0,52 |  |
| B-cells (CD19) | n | mean | median | SD | p-value |
| 0 mg/m^2^ | 47 | 10,96 | 9,97 | 5,28 | **0,02746** |
| ≥ 400 mg/m^2^ | 97 | 12,25 | 11,51 | 4,76 |  |
| Mature T-cells (CD3+) | n | mean | median | SD | p-value |
| 0 mg/m^2^ | 47 | 71,62 | 73,7 | 8,6 | 0,16614 |
| ≥ 400 mg/m^2^ | 97 | 70,13 | 69,04 | 8,3 |  |
| Helper T-lymphocytes | n | mean | median | SD | p-value |
| 0 mg/m^2^ | 47 | 41,78 | 42,16 | 7,51 | 0,87810 |
| ≥ 400 mg/m^2^ | 97 | 41,71 | 42,36 | 8,09 |  |
| Cytotoxic T-lymphocytes | n | mean | median | SD | p-value |
| 0 mg/m^2^ | 47 | 25,09 | 24,46 | 7,16 | 0,13986 |
| ≥ 400 mg/m^2^ | 97 | 23,57 | 22,84 | 8,59 |  |
| NKT cells | n | mean | median | SD | p-value |
| 0 mg/m^2^ | 47 | 5,47 | 4,09 | 4,296 | 0,27821 |
| ≥ 400 mg/m^2^ | 97 | 4,98 | 3,49 | 4,96 |  |
| NK cells | n | mean | median | SD | p-value |
| 0 mg/m^2^ | 47 | 15,35 | 13,71 | 7,48 | 0,53531 |
| ≥ 400 mg/m^2^ | 97 | 15,899 | 14,84 | 7,48 |  |
| Regulatory T -cells | n | mean | median | SD | p-value |
| 0 mg/m^2^ | 47 | 8,27 | 8,45 | 2,05 | 0,48790 |
| ≥ 400 mg/m^2^ | 95 | 8,11 | 7,87 | 2,10 |  |
| Classic monocytes | n | mean | median | SD | p-value |
| 0 mg/m^2^ | 39 | 80,35 | 81,52 | 5,25 | 0,68449 |
| ≥ 400 mg/m^2^ | 87 | 79,9 | 80,18 | 5,9 |  |
| Intermediate monocytes | n | mean | median | SD | p-value |
| 0 mg/m^2^ | 39 | 4,43 | 3,53 | 2,55 | 0,37312 |
| ≥ 400 mg/m^2^ | 85 | 4,46 | 4,13 | 2,47 |  |
| Nonclassical monocytes | n | mean | median | SD | p-value |
| 0 mg/m^2^ | 39 | 9,53 | 8,39 | 4,42 | 0,52192 |
| ≥ 400 mg/m^2^ | 85 | 9,84 | 8,73 | 4,45 |  |
| PMN -MDSC | n | mean | median | SD | p-value |
| 0 mg/m^2^ | 22 | 0,49 | 0,335 | 0,53 | 0,95117 |
| ≥ 400 mg/m^2^ | 44 | 0,49 | 0,36 | 0,68 |  |
| M-MDSC ( % total monocytes) | n | mean | median | SD | p-value |
| 0 mg/m^2^ | 22 | 6,03 | 5,18 | 3,33 | 0,85430 |
| ≥ 400 mg/m^2^ | 44 | 6,07 | 5,41 | 3,24 |  |
| PMN ( % total leukocytes) | n | mean | median | SD | p-value |
| 0 mg/m^2^ | 22 | 0,135 | 0,065 | 0,23 | 0,70792 |
| ≥ 400 mg/m^2^ | 44 | 0,13 | 0,08 | 0,16 |  |
| M-MDSC ( % total leukocytes) | n | mean | median | SD | p-value |
| 0 mg/m^2^ | 22 | 3,99 | 3,465 | 2,45 | 0,90795 |
| ≥ 400 mg/m^2^ | 44 | 3,89 | 3,8 | 2,48 |  |
| Conventional DC HLADR+ lin- | n | mean | median | SD | p-value |
| 0 mg/m^2^ | 45 | 76,49 | 80,19 | 10,82 | 0,41748 |
| ≥ 400 mg/m^2^ | 88 | 78,49 | 79,21 | 9,37 |  |
| Plasmacytoid DC HLADR+ lin- | n | mean | median | SD | p-value |
| 0 mg/m^2^ | 33 | 11,65 | 11,06 | 5,55 | 0,66638 |
| ≥ 400 mg/m^2^ | 70 | 11,16 | 10,77 | 5,11 |  |
| cDC ( % total leukocytes) | n | mean | median | SD | p-value |
| 0 mg/m^2^ | 45 | 1,07 | 0,92 | 0,51 | 0,82127 |
| ≥ 400 mg/m^2^ | 88 | 1,06 | 0,97 | 0,45 |  |
| pDC ( % total leukocytes) | n | mean | median | SD | p-value |
| 0 mg/m^2^ | 33 | 0,14 | 0,15 | 0,05 | 0,55906 |
| ≥ 400 mg/m^2^ | 70 | 0,14 | 0,13 | 0,07 |  |
| CD16+ DC  lin- HLADR+ | n | mean | median | SD | p-value |
| 0 mg/m^2^ | 28 | 56,62 | 57,38 | 15,27 | 0,46678 |
| ≥ 400 mg/m^2^ | 44 | 60,16 | 60,935 | 13,14 |  |
| DC2 (% DC) | n | mean | median | SD | p-value |
| 0 mg/m^2^ | 37 | 16,43 | 17,25 | 6,04 | 0,24157 |
| ≥ 400 mg/m^2^ | 77 | 15,23 | 14,97 | 5,65 |  |
| Immunoregulatory index (CD4+/CD8+) | n | mean | median | SD | p-value |
| 0 mg/m^2^ | 46 | 1,87 | 1,72 | 0,76 | 0,34614 |
| ≥ 400 mg/m^2^ | 97 | 2,22 | 1,85 | 1,13 |  |
| DNT- cells (CD3+CD8-CD4-) | n | mean | median | SD | p-value |
| 0 mg/m^2^ | 47 | 4,74 | 3,77 | 3,36 | 0,74125 |
| ≥ 400 mg/m^2^ | 97 | 4,85 | 4,06 | 3,55 |  |

**Supplementary Table 8: Comparison of the group at administration of less than 400 mg/m2 and at administration of more than 400 mg/m2**

| Neutrophils | n | mean | median | SD | p-value |
| --- | --- | --- | --- | --- | --- |
| ≤ 400 mg/m^2^ | 56 | 59,4 | 59,715 | 8,496 | 0,44989 |
| ≥ 400 mg/m^2^ | 97 | 60,55 | 60,45 | 8,84 |  |
| Lymphocytes | n | mean | median | SD | p-value |
| ≤ 400 mg/m^2^ | 56 | 29,67 | 28,95 | 7,22 | 0,53950 |
| ≥ 400 mg/m^2^ | 97 | 28,67 | 28,41 | 8,26 |  |
| Monocytes | n | mean | median | SD | p-value |
| ≤ 400 mg/m^2^ | 56 | 8,98 | 8,8 | 2,16 | 0,91704 |
| ≥ 400 mg/m^2^ | 97 | 9,00 | 8,71 | 2,57 |  |
| Eosinophils | n | mean | median | SD | p-value |
| ≤ 400 mg/m^2^ | 56 | 3,02 | 2,84 | 1,73 | **0,00025** |
| ≥ 400 mg/m^2^ | 97 | 2,14 | 1,66 | 1,73 |  |
| Basophils | n | mean | median | SD | p-value |
| ≤ 400 mg/m^2^ | 56 | 0,75 | 0,63 | 0,42 | 0,54689 |
| ≥ 400 mg/m^2^ | 97 | 0,71 | 0,62 | 0,52 |  |
| B-cells (CD19) | n | mean | median | SD | p-value |
| ≤ 400 mg/m^2^ | 56 | 11,64 | 11,54 | 3,43 | 0,52335 |
| ≥ 400 mg/m^2^ | 97 | 12,25 | 11,51 | 4,76 |  |
| Mature T-cells (CD3+) | n | mean | median | SD | p-value |
| ≤ 400 mg/m^2^ | 56 | 72,14 | 72,395 | 5,83 | 0,11468 |
| ≥ 400 mg/m^2^ | 97 | 70,13 | 69,04 | 8,3 |  |
| Helper T-lymphocytes | n | mean | median | SD | p-value |
| ≤ 400 mg/m^2^ | 56 | 41,16 | 42,23 | 8,66 | 0,90353 |
| ≥ 400 mg/m^2^ | 97 | 41,71 | 42,36 | 8,09 |  |
| Cytotoxic T-lymphocytes | n | mean | median | SD | p-value |
| ≤ 400 mg/m^2^ | 56 | 24,22 | 22,76 | 6,55 | 0,56098 |
| ≥ 400 mg/m^2^ | 97 | 23,57 | 22,84 | 8,59 |  |
| NKT cells | n | mean | median | SD | p-value |
| ≤ 400 mg/m^2^ | 56 | 5,23 | 4,29 | 4,41 | 0,41546 |
| ≥ 400 mg/m^2^ | 97 | 4,98 | 3,49 | 4,96 |  |
| NK cells | n | mean | median | SD | p-value |
| ≤ 400 mg/m^2^ | 56 | 14,28 | 13,62 | 5,92 | 0,24879 |
| ≥ 400 mg/m^2^ | 97 | 15,899 | 14,84 | 7,48 |  |
| Regulatory T -cells | n | mean | median | SD | p-value |
| ≤ 400 mg/m^2^ | 56 | 8,445 | 8,43 | 2,19 | 0,40863 |
| ≥ 400 mg/m^2^ | 97 | 8,11 | 7,87 | 2,10 |  |
| Classic monocytes | n | mean | median | SD | p-value |
| ≤ 400 mg/m^2^ | 48 | 78,07 | 78,44 | 5,39 | 0,06529 |
| ≥ 400 mg/m^2^ | 87 | 79,9 | 80,18 | 5,91 |  |
| Intermediate monocytes | n | mean | median | SD | p-value |
| ≤ 400 mg/m^2^ | 48 | 4,17 | 3,96 | 2,09 | 0,29289 |
| ≥ 400 mg/m^2^ | 85 | 4,62 | 4,13 | 2,47 |  |
| Nonclassical monocytes | n | mean | median | SD | p-value |
| ≤ 400 mg/m^2^ | 48 | 10,55 | 10,48 | 3,97 | 0,10401 |
| ≥ 400 mg/m^2^ | 85 | 9,84 | 8,73 | 4,45 |  |
| PMN -MDSC | n | mean | median | SD | p-value |
| ≤ 400 mg/m^2^ | 29 | 0,52 | 0,26 | 0,53 | 0,76507 |
| ≥ 400 mg/m^2^ | 44 | 0,49 | 0,36 | 0,68 |  |
| M-MDSC ( % total monocytes) | n | mean | median | SD | p-value |
| ≤ 400 mg/m^2^ | 29 | 5,72 | 5,06 | 3,07 | 0,84802 |
| ≥ 400 mg/m^2^ | 44 | 6,07 | 5,41 | 3,24 |  |
| PMN ( % total leukocytes) | n | mean | median | SD | p-value |
| ≤ 400 mg/m^2^ | 29 | 0,13 | 0,07 | 0,18 | 0,79940 |
| ≥ 400 mg/m^2^ | 44 | 0,126 | 0,08 | 0,16 |  |
| M-MDSC ( % total leukocytes) | n | mean | median | SD | p-value |
| ≤ 400 mg/m^2^ | 29 | 4,16 | 3,82 | 2,29 | 0,55771 |
| ≥ 400 mg/m^2^ | 44 | 3,89 | 3,8 | 2,48 |  |
| Conventional DC HLADR+ lin- | n | mean | median | SD | p-value |
| ≤ 400 mg/m^2^ | 51 | 78,29 | 79,9 | 9,69 | 0,93209 |
| ≥ 400 mg/m^2^ | 88 | 78,49 | 79,21 | 9,37 |  |
| Plasmacytoid DC HLADR+ lin- | n | mean | median | SD | p-value |
| ≤ 400 mg/m^2^ | 35 | 11,63 | 11,22 | 5,47 | 0,67591 |
| ≥ 400 mg/m^2^ | 70 | 11,16 | 10,77 | 5,11 |  |
| cDC ( % total leukocytes) | n | mean | median | SD | p-value |
| ≤ 400 mg/m^2^ | 51 | 1,13 | 1,06 | 0,42 | 0,27359 |
| ≥ 400 mg/m^2^ | 88 | 1,06 | 0,97 | 0,45 |  |
| pDC ( % total leukocytes) | n | mean | median | SD | p-value |
| ≤ 400 mg/m^2^ | 36 | 0,15 | 0,145 | 0,06 | 0,20890 |
| ≥ 400 mg/m^2^ | 70 | 0,14 | 0,13 | 0,07 |  |
| CD16+ DC  lin- HLADR+ | n | mean | median | SD | p-value |
| ≤ 400 mg/m^2^ | 30 | 58,73 | 59,66 | 11,10 | 0,59336 |
| ≥ 400 mg/m^2^ | 44 | 60,16 | 60,94 | 13,14 |  |
| DC2 (% DC) | n | mean | median | SD | p-value |
| ≤ 400 mg/m^2^ | 43 | 18,57 | 15,32 | 10,71 | 0,24042 |
| ≥ 400 mg/m^2^ | 77 | 15,23 | 14,97 | 5,65 |  |
| Immunoregulatory index (CD4+/CD8+) | n | mean | median | SD | p-value |
| ≤ 400 mg/m^2^ | 56 | 1,86 | 1,78 | 0,75 | 0,46018 |
| ≥ 400 mg/m^2^ | 97 | 2,11 | 1,85 | 1,13 |  |
| DNT- cells (CD3+CD8-CD4-) | n | mean | median | SD | p-value |
| ≤ 400 mg/m^2^ | 56 | 6,76 | 5,27 | 6,68 | **0,01839** |
| ≥ 400 mg/m^2^ | 97 | 4,85 | 4,06 | 3,55 |  |

**Supplementary figure 1: Gating strategy**

**Supplementary figure 2: Gating strategy**
